# Low Predictability of Colour Polymorphism in Introduced Guppy (*Poecilia reticulata*) Populations in Panama

**DOI:** 10.1371/journal.pone.0148040

**Published:** 2016-02-10

**Authors:** Celestino Martínez, Carmen Chavarría, Diana M. T. Sharpe, Luis Fernando De León

**Affiliations:** 1 Centro de Biodiversidad y Descubrimiento de Drogas, Instituto de Investigaciones Científicas y Servicios de Alta Tecnología (INDICASAT-AIP), Panamá, República de Panamá; 2 Department of Biology, McGill University, Montréal, Quebec, Canada; University of Lausanne, SWITZERLAND

## Abstract

Colour polymorphism is a recurrent feature of natural populations, and its maintenance has been studied in a range of taxa in their native ranges. However, less is known about whether (and how) colour polymorphism is maintained when populations are removed from their native environments, as in the case of introduced species. We here address this issue by analyzing variation in colour patterns in recently-discovered introduced populations of the guppy (*Poecilia reticulata*) in Panama. Specifically, we use classic colour analysis to estimate variation in the number and the relative area of different colour spots across low predation sites in the introduced Panamanian range of the species. We then compare this variation to that found in the native range of the species under low- and high predation regimes. We found aspects of the colour pattern that were both consistent and inconsistent with the classical paradigm of colour evolution in guppies. On one hand, the same colours that dominated in native populations (orange, iridescent and black) were also the most dominant in the introduced populations in Panama. On the other, there were no clear differences between either introduced-low and native low- and high predation populations. Our results are therefore only partially consistent with the traditional role of female preference in the absence of predators, and suggest that additional factors could influence colour patterns when populations are removed from their native environments. Future research on the interaction between female preference and environmental variability (e.g. multifarious selection), could help understand adaptive variation in this widely-introduced species, and the contexts under which variation in adaptive traits parallels (or not) variation in the native range.

## Introduction

Colour polymorphism is a common feature of natural populations, and one that is critical for understanding adaptive evolution in nature [1–5]. Colour polymorphism is often linked to adaptive divergence [6], and is thought to be involved in adaptive radiation across taxa such as fish [7], birds [8], insects [9] and molluscs [10]. Furthermore, the stochastic and selective forces (e.g. sexual and natural selection) maintaining colour polymorphism, and its association with fitness have been widely explored in natural populations [11–14]. A less explored aspect of colour polymorphism is whether (and how) colour patterns are maintained when species are faced with novel environments. Introduced species provide an excellent model to test this question because they often face novel abiotic conditions, and experience novel ecological interactions with predators, prey, competitors and/or parasites with which they have no shared evolutionary history [15]. Under such conditions, stochastic or selective regimes might be quite different from those experienced in the native range of the species, and moreover, the phenotypic response (i.e. color) might not always parallel that observed in the native range of the species. On the other hand, if environmental conditions in the introduced range of the species are similar to those experienced in its native range, we might observe colour patterns to be consistent with those of the native range of the species.

Predictable colour polymorphisms (i.e., the maintenance of native colour patterns in the introduced range) would suggest that patterns of mate choice and/or predation are consistent across native and introduced ranges, hence supporting the role of natural and/or sexual selection in driving colour variation in nature [12,16]. In contrast, divergent colour polymorphisms would suggest that additional local abiotic factors such as turbidity or nutrient availability [17,18] may be influencing colour polymorphism in introduced populations.

We here address this issue by analyzing the colour patterns of recently-discovered introduced populations of the guppy (*Poecilia reticulata*) in Panama. Specifically, we ask: what are the patterns of colour variation across sites in the introduced Panamanian range of the species? And, how does colour variation in introduced populations compare to that in the native range of the species under low- and high predation regimes?

The guppy (*Poecilia reticulata* Peters, 1859), is a widespread freshwater fish species native to northeastern South America, including Venezuela, the Margarita and Caribe islands, Barbados, Trinidad, and Tobago [16,19,20]. One of the most striking features of this species is its marked sexual dimorphism, with females being larger and drabber than males. The latter typically display multiple colour spots constituted mainly by carotenoids and pteridines (red, orange and yellow), melanin (black) and structural colours (blue, iridescent, green and silver) [12,21,22]. These colour spots tend to vary among populations in their position, size, and number [23–25].

Given this extreme colour polymorphism, *P*. *reticulata* has become an important model for the study of adaptive evolution in nature [12,16,26]. Colour polymorphism observed in male guppies is mainly the result of a balance between natural and sexual selection in the wild [12,16,21,27–29]. That is, female preference is biased toward highly conspicuous males, but the same time, more conspicuous males are more visible to predators [16,30–32]. This interplay between female preference and predation generates predictable colour patterns in different environments across the native range of the species. Typically, in low predation sites in Trinidad, male guppies present a higher frequency of bright colours such as orange and iridescent [33–36], whereas in high predation sites, males tend to be drabber with a lower proportion of bright colours [33].

Although deviations from this pattern do exist (e.g., [37,38]), overall, these predictions have been repeatedly confirmed both in the wild [14,39] and in the laboratory [12,27,40]. However, it is less clear if these predictions hold when populations are introduced outside the native range of the species. Some examples include experimental introductions of guppies from high- to low-predation sites [12,41,42], but these introductions have still been restricted to sites within the native range of the species.

*Poecilia reticulata* has been introduced worldwide, and viable populations have been found in a wide range of environments [43]. Introduced guppy populations are often the result either of intentional stocking for mosquito control or accidental releases from the aquarium trade (reviewed in [43]). Despite this broad global distribution, only a handful of studies have looked at colour variation in introduced populations: in Australia (e.g., [44–46]) and in South Africa (e.g., [31,47]). These studies have generally confirmed the traditional view of the role of female preference in driving male colour patterns. However, one recent study suggested that female preference and male couloration might not always respond to selection as expected from classical theory, suggesting that additional factors are involved, including low heritability, and constraints from genetic and environmental covariances [48]. Furthermore, Lindholm *et al*. [49], in an analysis of color variation between high-and low predation environments in Australia, showed that only one colour (orange area) was statistically different between introduced high-and low populations [49]. Together, these findings raise the possibility that colour polymorphism in introduced populations of *P*. *reticulata* might not always be consistent with what it is expected from the native range of the species. One way to inform this possibility is to explore colour patterns in other parts of the introduced range of the species, and to contrast those patterns with those of the native range of the species. Despite the ubiquity of *P*. *reticulata*, to our knowledge such comparisons have not been previously made. We here explore this possibility by examining colour variation in recently discovered introduced populations of *P*. *reticulata* in Panama. Although this work is exploratory, we hope to stimulate further analyses of colour variation in other areas of the introduced range of the species.

In Panama, *P*. *reticulata* was introduced for mosquito control in the Panama Canal Zone between 1912 and 1914 [50]. It is reported that, as part of this campaign, a few individuals from Barbados were “bred in captivity”, and that “batches were liberated in several places now included in Gatun Lake” [50]. However, subsequent surveys did not find any individuals in the lake, implying that the species failed to establish [50]. Later, Loftin [51] collected one individual of *P*. *reticulata* from the Corona River in the Panama Canal Zone but suggested that the specimen had likely been recently released from a nearby aquarium [51]. No further records of *P*. *reticulata* were reported in Panama until 2004, when Gonzalez [52] reported individuals of the species in Los Chorros River in Santiago, Veraguas (reviewed in [53])

**Fig 1:**
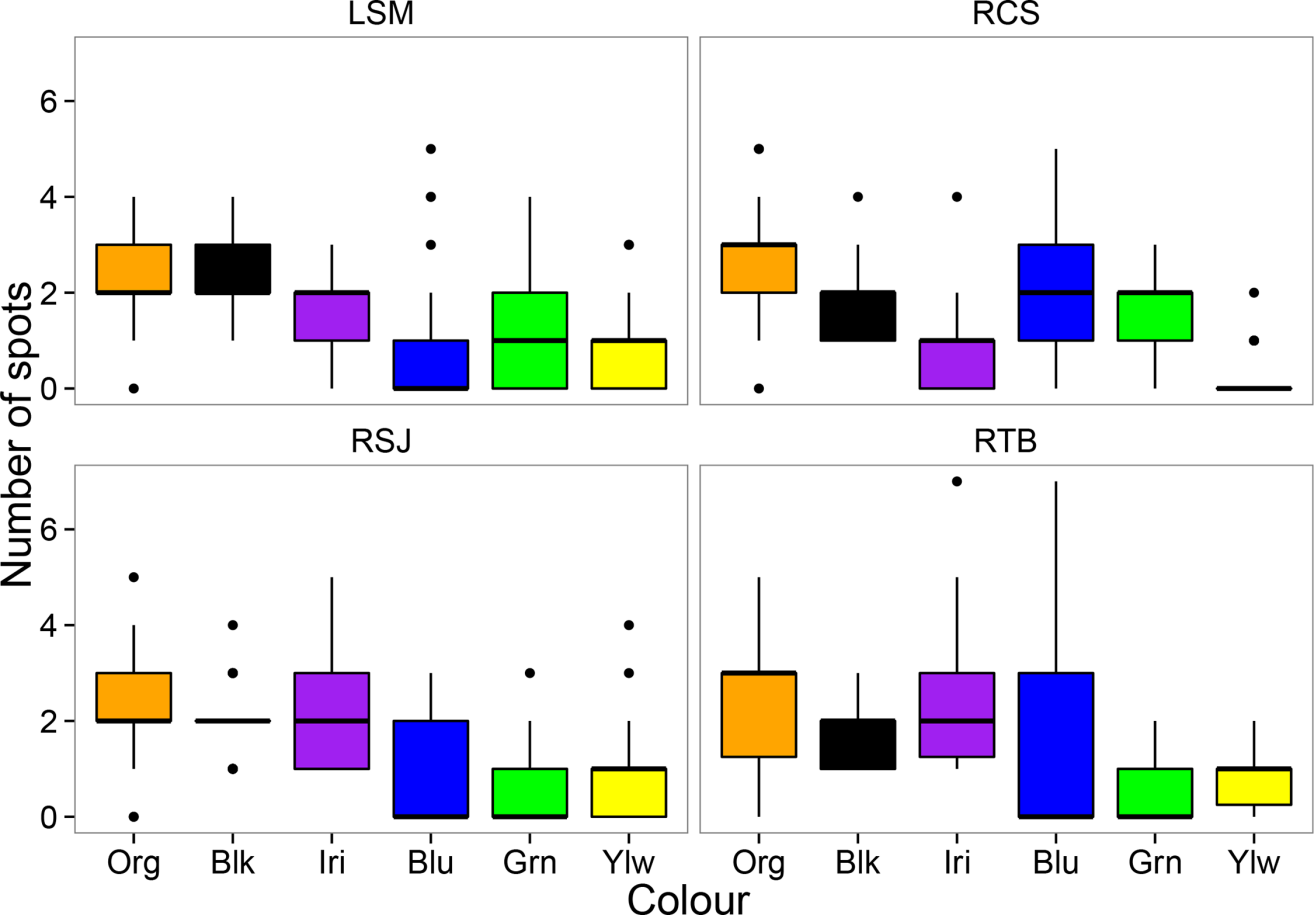
Number of colour spots in introduced populations of *P*. *reticulata* in Panama. Sampling sites are: Lake Santa Mónica (LSM), Cristo Sacramentado (RCS), San Juan (RSJ) and Tres Brazos (RTB). Boxes represent the interquartile range and the error bars represent the total range of the data. Colour conventions are: black (Blk), orange (Org), iridescent (Iri), green (Grn), blue (Blu) and yellow (Ylw).

Herein, we confirm this report with the use of DNA barcoding, and report two more populations in the Santiago area, and one in Central Panama (Fig 1). Furthermore, we take advantage of these guppy populations to explore colour patterns in introduced populations in Panama, and to examine how these patterns compare to those in the native range of the species. Specifically, we analyze variation in the area and number of different colour spots across sites, and compare these colour phenotypes to those of populations from low and high predation sites in the native range of the species. Our introduced populations can all be considered as existing in low predation environments because previous surveys (reviewed in [53]), and our own observations suggest that piscivores are absent from the sites colonized by introduced guppies in Panama.

## Methods

### Statement of ethics

This research was approved by the Institutional Animal Care and Use Committee (IACUC) at the Instituto de Investigaciones Científicas y Servicios de Alta Tecnología (INDICASAT-AIP). To minimize stress while photographs were taken, fish were anesthetized with a dose of eugenol (C_10_H_12_O_2_) derived from clove oil. The sampling permit was obtained from Autoridad Nacional del Ambiente de Panamá.

### Sampling sites

Fish were collected in the dry season (February-April 2013) at three sites in the Province of Veraguas and one site in the Province of Panama (Table 1). The three Veraguas sites were San Juan River, Cristo Sacramentado stream, and Santa Monica Lake, all located in the outskirts of the city of Santiago. The site in the province of Panama was Tres Brazos stream located in the municipality of Pacora (Table 1).

**Table 1:** Environmental and geographic parameters of the sampling sites in Panama.

Sampling site	Oxygen (mg/L)	Temperature (°C)	Description	Coordinates	
**San Juan (RSJ)**	0.8	24.9	Natural urban stream	08° 06' 40.4''N	80° 58' 49.0''W
**Santa Mónica(LSM)**	2.0	29.7	Small lake surrounded by urban zone	08° 05' 18.5'' N	80° 58' 31.3''W
**Cristo Sacramentado (RCS)**	0.1	29.5	Highly-polluted urban ditch	08° 05' 38.3'' N	80° 58' 52.3''W
**Tres Brazos (RTB)**	0.9	27.1	Seasonal rural stream	09° 14' 01.0'' N	79° 15' 17.9''W

These sites were selected because they present stable populations of *P*. *reticulata*, either based on previous reports (i.e., San Juan [52]), or our own exploratory sampling (Lake Santa Monica, Tres Brazos, Cristo Sacramentado). These sites all have a simple fish community and completely lack piscivores [53], possibly due to high levels of water pollution and low levels of dissolved oxygen (Table 1). The only fish species observed during our visual surveys, using dip-nets and removing the shore vegetation and root masses, were *Astyanax ruberrimus* and *Andinoacara coeruleopunctatus* in the San Juan River, and Tilapia (*Oreochromis niloticus*) in Santa Monica Lake. At the other sites only *P*. *reticulata* was observed. The two species observed in San Juan River (*A*. *ruberrimus* and *A*. *coeruleopunctatus*) feed only on insects, fruits and seeds [54]. Furthermore, our recent stomach content analysis of 250 specimens of *Astyanax ruberimus* from several streams in Panama revealed that the diet of this species is exclusively composed of insects, seeds and detritus (De León et al. Unpublished data).

### Species identification

We obtained tissue samples from 5 individuals from each study site, and extracted total DNA using a standard QiagenTM extraction kit. A standard sequencing protocol [55] was used to amplify the full-length (658 bp) of the cytochrome c oxidase subunit I (COI) barcode region using the primers LCO1490 and HCO2198 [56]. To confirm the species identity we performed BLAST searches for publicly available sequences in GeneBank. From these searchers we obtained all sequences with 98% similarity, and built a Neighbour-joining tree (NJ) using Kimura-2-parameter (K2P) distances as implemented in the software MEGA5.0 (www.megasoftware.net). Bootstrap support for this analysis was obtained after 1000 replicates.

### Colour analysis

Using a hand-net, we collected between 28–30 males of *P*. *reticulata* across sites. Fish were transported to the laboratory, where they were processed following the methods of Millar and colleagues [57]. Fish were anesthetized with a dose of eugenol (C_10_H_12_O_2_) derived from clove oil following recommendations from Gray and colleagues [58].

Lateral images of the left side of each fish were taken with a digital camera (CANON xti) fitted with a 60mm macro lens and placed at a standard distance with the aid of a tripod. Each individual was placed on a white background with a millimetric scale, and colour standards (Red, Orange, Yellow, Blue, Green, White and Black). To ensure the accuracy of the colours of each individual, the photos were taken using full spectrum white light (5500K - 120V). Using the software Image J (version 1.45g), we measured standard length, body area (excluding the fins and tail), and the area and the number of each colour spot (excluding the fins and tail). All images were analyzed by the same person (CM).

The most common colour spots were classified into the colour categories described by Endler [11] and Millar *et al*. [57]. These categories included: orange (which also included red), black (which included black fuzzy), yellow, blue (which included purple), green, and iridescent (which included blue-violet, green-bronze and silver). For each colour category we calculated three main variables: the total number of spots, total area of each spot, and the total relative area (total area covered by that colour divided by the total body area of the fish).

Finally, to estimate the consistency of colour patterns in the introduced population relative to those in the native range of the species, we compared our results to data reported in the literature for native populations. For this comparison we focused on two colour parameters (total number of spots and relative area) previously estimated for native populations [12,13,29,38,41,42,57]. We only included data from studies with roughly similar methodology to ours (Table 2). The main criterion for inclusion was that colour parameters had been reported individually. The number of colour spots needed to be reported as the absolute number of spots independent of body size, and the area of colour spots as the relative area of each spot divided by the body area and excluding fins. We also attempted to incorporate only colour measurements estimated from wild native populations.

**Table 2:** Colour patterns in populations of *Poecilia reticulata* from native low- and introduced low- and high predation environments. The data represent the mean ± standard deviation (SD) of the number and the relative area of the most commonly reported colour spots in the literature and in the present study. N represents the number of estimates of colour parameters at population level obtained from the literature (Native) and from this study (Panama) at both low (LP) and high (HP) predation regimes. Total refers to the total number of populations across environments and colour parameters. The complete list of values and authors of each study can be found in S2 Table.

Colour	Origin	Predation	Spot Number		Relative Area		Total
			Mean	N	Mean	N	
Black	Native	LP	2.60 ± 0.84	8	0.15 ± 0.10	14	22
		HP	2.40 ± 0.88	10	0.12 ± 0.09	13	23
	Panama	LP	1.99 ± 0.17	4	0.13 ± 0.03	4	8
Blue	Native	LP	1.85 ± 1.23	5	0.06 ± 0.01	4	9
		HP	1.99 ± 0.81	7	0.07 ± 0.04	3	10
	Panama	LP	1.34 ± 0.61	4	0.04 ± 0.03	4	8
Iridescent	Native	LP	3.00 ± N/A	1	0.11 ± 0.05	3	4
		HP	1.60 ± 1.06	3	0.13 ± 0.07	3	6
	Panama	LP	1.83 ± 0.70	4	0.08 ± 0.02	4	8
Orange	Native	LP	2.50 ± 0.72	8	0.11 ± 0.06	14	22
		HP	2.62 ± 0.82	10	0.09 ± 0.06	13	23
	Panama	LP	2.44 ± 0.15	4	0.08 ± 0.02	4	8
**Total**				68		83	151

These criteria excluded studies where populations derived from long-term laboratory experiments and where colour estimates included fin area and more advanced spectometry analyses (e.g., [37,41,59]). In addition, some studies focused their analysis on “biologically relevant” parameters (*sensu* [31,43]), where colour patterns are classified as carotenoid, melanic, and structural. We excluded data from studies where colours were combined [37,41] or where colour spots with low contribution were not reported [29]. Other studies reported colour data in an inaccessible format.

As mentioned above, our study in Panama focused exclusively on low predation environments. However, we did also compare our data to values previously reported for low- and high predation populations from the native range of the species.

### Data analysis

To quantify the variability in colour patterns within and among introduced populations of *P*. *reticulata* we estimated the coefficient of variation (CV), which represents the ratio of the standard deviation (*s*) to the mean (*x*) and is expressed as:

$$CV=\frac{s}{x}$$ (1)

We estimated this coefficient both within (*CV*_*w*_) and among (*CV*_*a*_) sites, and calculated the ratio *Cv*_*a*_*/CV*_*w*_ to assess the potential of population coding (PPC; [60,61]). Larger among population variability (PPC >1) would indicate a high degree of uniqueness (i.e., dominance) in colour pattern at each site [61].

To test for overall variation in colour patterns across sites, we performed two separate multivariate analysis of variance (MANOVA). The first included the number of spots of each colour as response variables, and site as the explanatory variable. The second included the relative areas of each colour as response variables, and site as the explanatory variable. When necessary, we square-root transformed the colour variables to satisfy the assumption of homogeneity of variances. To further explore individual colour differences across sites, we also performed separate ANOVAs with each colour (number of spots or relative area) as the response variable and site as the explanatory variable. To avoid potential bias due to violation of normality in the data, we repeated these analyses using nonparametric Kruskal-Wallis rank tests on the raw data.

To compare overall variation in colour patterns (number of spots and relative area) between introduced and native populations, we performed non-parametric (Kruskal-Wallis rank) tests with either the number of spots or the area of each colour as a function of environment (introduced vs. native). In this analysis we used mean values per population for the four colour categories (black, blue, iridescent, orange) most commonly reported in the literature [12,13,41,57]. For the analysis of colour spots, we did not include iridescent in our statistical analyses because only one study measured this colour. However, we did include it on the graphs for illustrative purposes. We did, however, include iridescent in the analysis of relative colour area. All analyses were performed in the software R, version 2.15 [62].

## Results

### Species identification

We obtained a total of 12 full COI sequences that showed little variation among individuals. For this reason, we used one consensus sequence for the subsequent analyses. Our BLAST searches successfully matched published sequences of *P*. *reticulata* with over 99% sequence similarity, confirming the identity of our samples. The NJ tree indicated that our samples fell well within other introduced populations of *P*. *reticulata* (e.g. Canada, Brazil, Cape Verde and India) previously reported in the literature (S1 Fig). Interestingly, there seemed to be some variation (>2% sequence divergence) between these introduced populations and the native population from Trinidad, which clusters with introduced populations from Indonesia; although the level of support for this clade is <50% (S1 Fig). All sequences have been submitted to GenBank (accession numbers: KT599862-KT599873).

### Colour patterns in introduced populations

**Fig 2:**
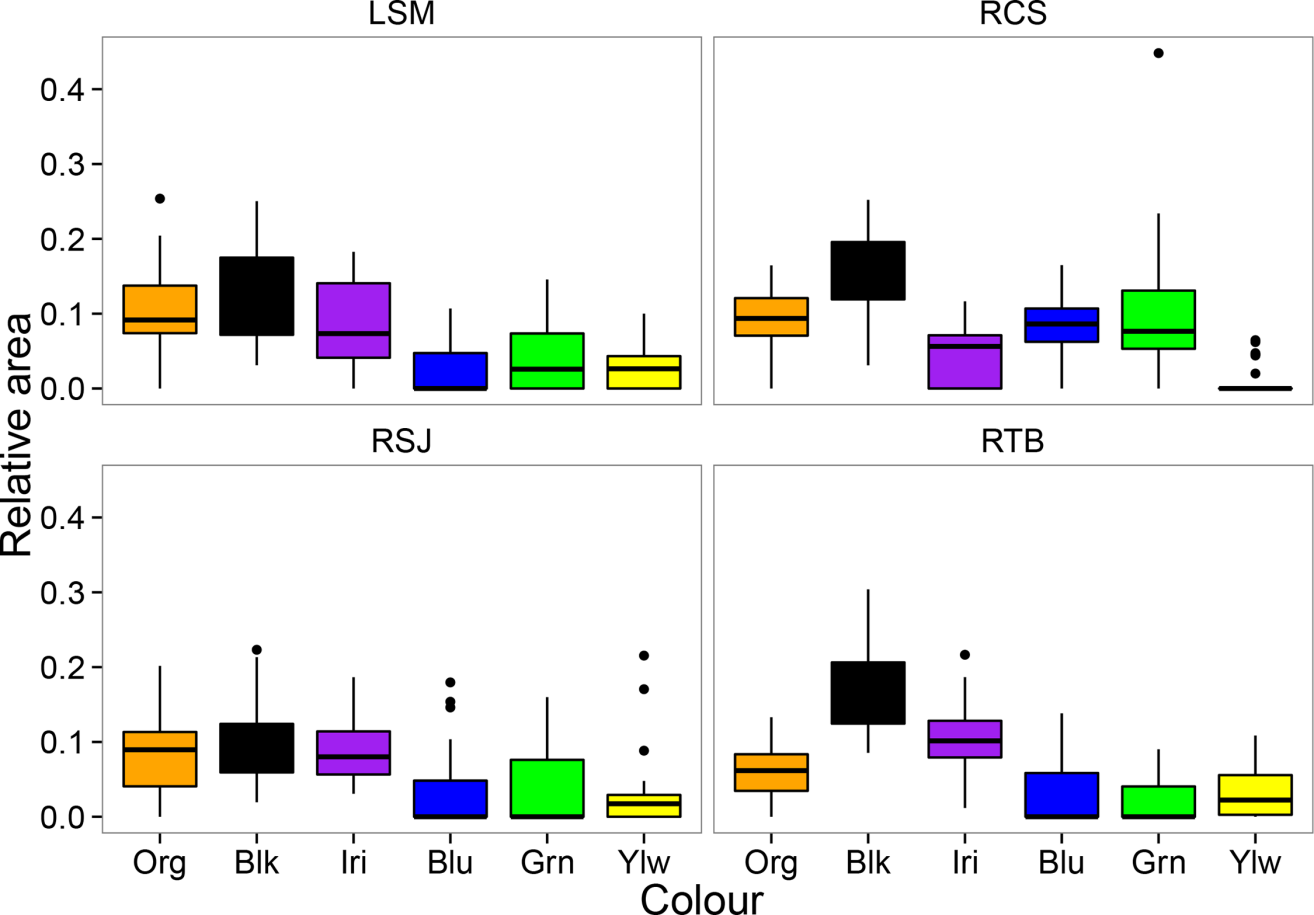
Relative area of different colour spots in introduced populations of *P*. *reticulata* in Panama. Boxes represent the interquartile range and the error bars represent the total range of the data. Site names and colour conventions are the same as in Fig 1.

MANOVA revealed significant overall differences in colour pattern across our four Panamanian sites, both in terms of the number (F_3,327_ = 4.7, p < 0.001) and relative area (F_3,327_ = 7.6, p < 0.001) of colour spots. Subsequent ANOVAs for each colour also showed considerable variation among sites, with most of the colours presenting statistically significant differences (Table 3). Similar results were obtained with the Kruskal-Wallis rank test (S1 Table). Nevertheless, the qualitative nature of colour patterns was broadly similar across the four introduced populations that we examined in Panama (Table 4). Orange, black and iridescent were the most important colours in three out of four sites, both in terms of the number of spots (Fig 1), and relative area (Fig 2). The remaining colours (blue, yellow and green) were generally less important (fewer spots, lower relative area), except in Cristo Sacramentado stream, where males tended to have a greater number of blue spots (Table 4).

**Table 3:** Color variation in introduced populations of *Poecilia reticulata*. Results from ANOVAs examining variation across sites in the number and relative area of colour spots in introduced populations of *P*. *reticulat*a from Panama. The analysis was based on 30 individuals per population. Values in bold represent statistically significant differences.

Colour	Number of spots			Relative area		
	Df	F	*P*	Df	F	*P*
**Black**	3, 112	1.38	0.251	3, 112	10.17	**<0.001**
**Iridescent**	3, 112	12.35	**<0.001**	3, 112	10.65	**<0.001**
**Orange**	3, 112	0.45	0.711	3, 112	5.09	**0.001**
**Yellow**	3, 112	4.79	**0.001**	3, 112	5.61	**0.001**
**Blue**	3, 112	5.08	**0.001**	3, 112	12.86	**0.001**
**Green**	3, 112	14.47	**<0.001**	3, 112	13.57	**<0.001**

**Table 4:** Colour parameters in introduced populations of *Poecilia reticulata* in Panama. The data represent mean ± standard deviation (SD) and samples sizes (N) estimated across sites: Lake Santa Mónica (LSM), Cristo Sacramentado (RCS), San Juan (RSJ) and Tres Brazos (RTB).

Parameter	Colour	LSM	RCS	RSJ	RTB
	Black	2.21±0.79 (28)	1.86±0.74 (29)	2.03±0.78 (29)	1.87±0.73 (30)
	Blue	0.93±1.44 (28)	2.14±1.06 (29)	0.79±1.11 (29)	1.50±2.01 (30)
Colour spot	Green	0.93±1.02 (28)	1.79±0.77 (29)	0.62±0.90 (29)	0.50±0.57 (30)
	Iridescent	1.61±0.74 (28)	0.97±0.87 (29)	2.17±1.04 (29)	2.57±1.48 (30)
	Orange	2.29±0.81 (28)	2.62±1.15 (29)	2.48±1.15 (29)	2.37±1.40 (30)
	Yellow	0.89±0.83 (28)	0.24±0.51 (29)	0.86±1.03 (29)	0.83±0.59 (30)
	Black	0.12±0.07 (28)	0.15±0.06 (29)	0.09±0.05 (29)	0.17±0.06 (30)
	Blue	0.03±0.03 (28)	0.08±0.04 (29)	0.03±0.05 (29)	0.03±0.05 (30)
Relative area	Green	0.04±0.04 (28)	0.10±0.09 (29)	0.04±0.05 (29)	0.02±0.02 (30)
	Iridescent	0.09±0.05 (28)	0.05±0.04 (29)	0.09±0.04 (29)	0.11±0.05 (30)
	Orange	0.11±0.06 (28)	0.09±0.04 (29)	0.08±0.05 (29)	0.06±0.04 (30)
	Yellow	0.03±0.03 (28)	0.01±0.02 (29)	0.03±0.05 (29)	0.04±0.04 (30)

**Fig 3:**
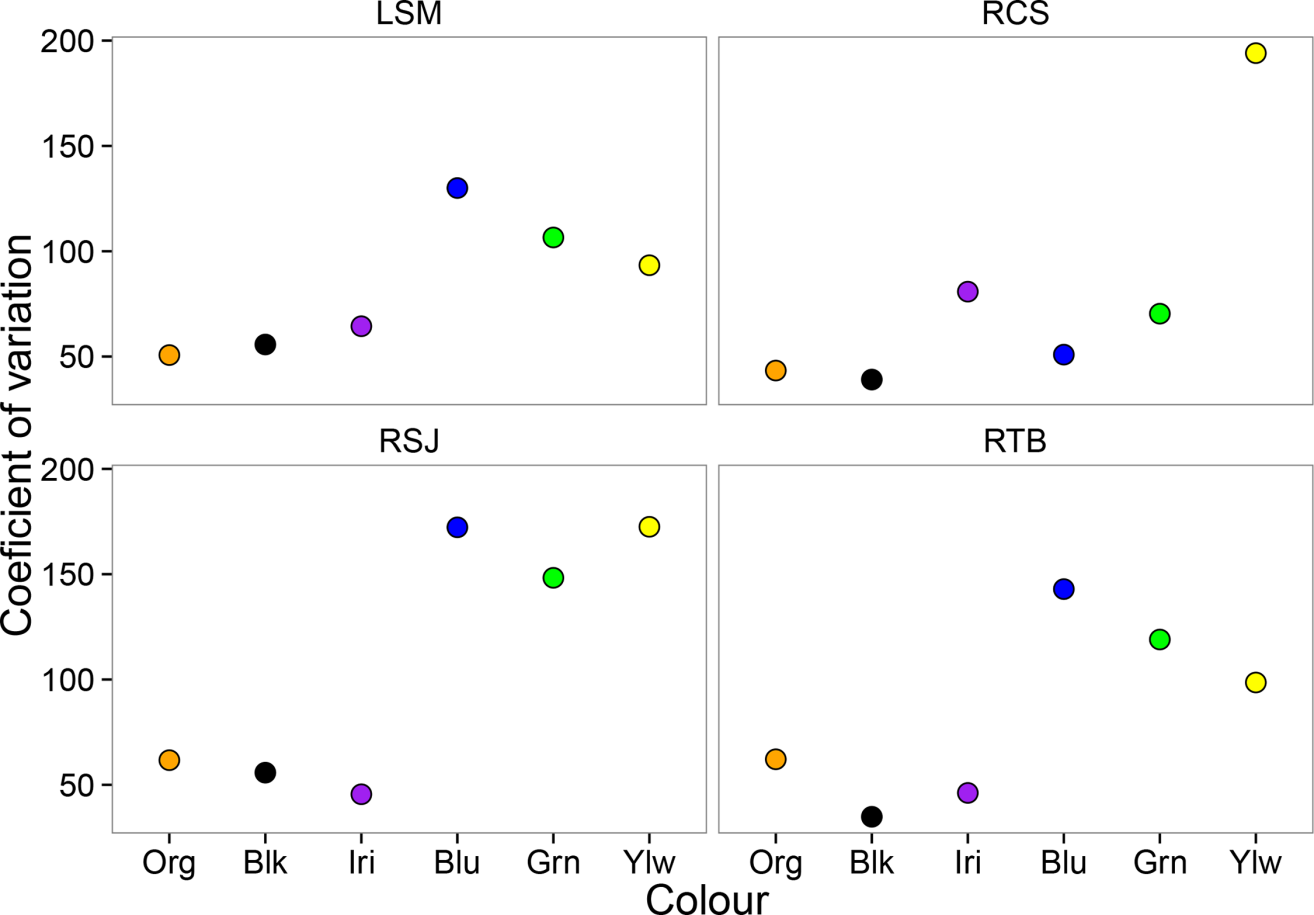
Variability in colour patterns in introduced populations of *P*. *reticulata* in Panama. Variability is given by the coefficient of variation (CV) across sites. Colour conventions are the same as in Fig 1.

The coefficient of variation (CV) showed considerable variability both within and among sites. Interestingly, the colours with the highest relative area at all sites (black, orange and iridescent) were also the least variable (lower CV), whereas the less abundant colours (yellow, blue and green) were the most variable (high CV) (Fig 3). Furthermore, the only colours with PPC values >1 were black (1.92), orange (1.64) and iridescent (1.51), suggesting that these three colours could represent the most distinct features of the guppy colour polymorphism across sites.

### Colour patterns in introduced vs. native populations

**Fig 4:**
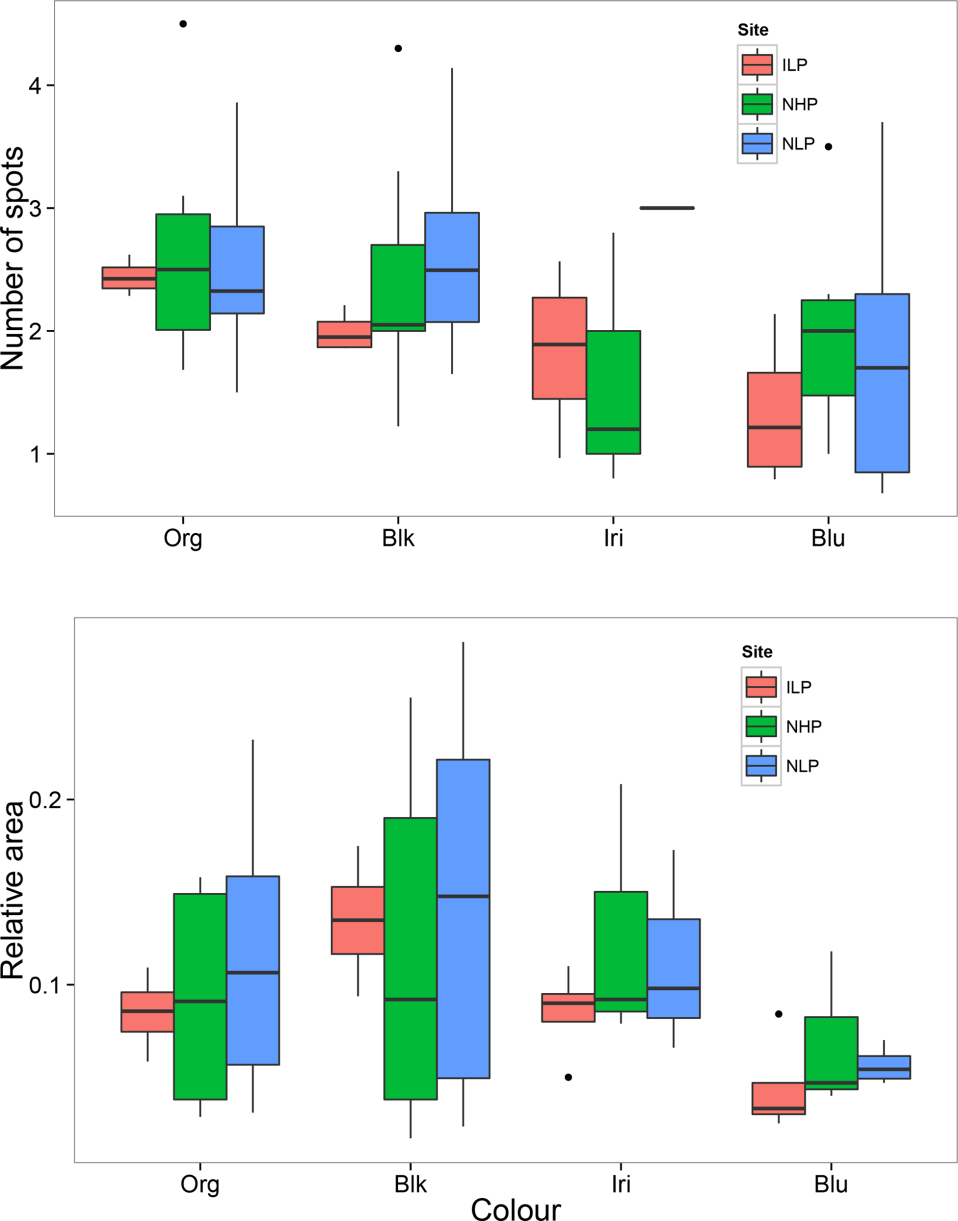
Color variation between native and introduced populations of *P*. *reticulata*. The data represent number of spots (top panel) and relative colour area (bottom panel) in introduced low predation populations (ILP), and native low (NLP) and high (NHP) predation populations. Boxes represent the interquartile range and the error bars represent the total range of the data. Data from native populations were obtained from the published literature (S2 Table).

We compiled published population-level estimates of colour patterns in *Poecilia reticulata* from 7 studies that reported data from low and high predation environments in the native range of the species. Of these 7 studies, all reported relative area, but only a subset reported spot number as well. Also, not all studies examined all colours, so sample sizes for some colours were lower: exact sample sizes for each colour and each parameter are given in Table 2 and S2 Table. When comparing colour patterns between introduced (present study, *N* = 4 for both area and number of spots) and native populations (Table 2, *N* = 14 for area, and *N* = 8 for number of spots) we found very similar patterns across populations overall. There were no significant differences in either the number of spots (all colours, *P* > 0.23) or relative area (all colours, *P* > 0.25) between introduced and native populations for any colour in low predation environments (Fig 4). Similarly, we found no significant differences in the number of spots (all colours, *P* > 0.16) and relative area (all colours, *P* > 0.16) between introduced and native populations in high predation environments (Fig 4). Finally, when comparing colour variation across our data set, we observed that introduced populations were intermediate and overlapping with both native low and high populations. In addition, there were no statistical differences between native low- and high predation populations for either number of spots (all colours, *P* > 0.18) or colour area (all colours, *P* > 0.22) (Fig 4).

## Discussion

We here take advantage of recently-discovered introduced populations of *P*. *reticulata* in Panama to explore how colour patterns vary across introduced populations, and how these patterns fit the expected colour variation typically observed in low- and high predation environments in the native range of the species. We discuss each of these findings in turn, before addressing some of the potential limitations of this study, and the broader implications for the study of colour polymorphism and adaptation during biological invasions.

### Colour patterns in introduced populations

We found that both number of spots and relative area varied consistently across sites in the introduced range of species in Panama. Specifically, the most abundant colour categories (orange, iridescent and black) were less variable, and seemed to show some degree of fixation (PPC >1) across introduced populations. In contrast, the least abundant colours categories (green, blue, and yellow) were more variable across sites, and did not show evidence of fixation (PPC<1) at any of the sites. In both cases, however, we found some significant differences across sites, supporting previous studies that show extreme colour variation in *P*. *reticulata* from native [63] and introduced environments [48,64].

### Colour patterns in introduced vs. native populations

When comparing the colour patterns of introduced (Panama) and native (Trinidad) populations, we found features that were both consistent and inconsistent with the classical paradigm of colour evolution in guppies. On one hand, the same colours (orange, iridescent and black) that dominate in native populations [11–14] were also the most dominant in our introduced populations in Panama. This supports the traditional view that certain colour ornaments are universally favored in *P*. *reticulata*, even when populations are far removed from their native environment.

Theory, as well as previous empirical work, suggests that in the absence of predators, female preference should repeatedly drive the evolution of conspicuous colour patterns in male guppies [12,14,65]. Specifically, females from native populations in low-predation environments typically show strong preference for orange [16,30,33], black [65] and iridescent colouration [32], suggesting that this pattern of female preference may be highly conserved, possibly through the use of an universal set of colours as honest signals for male quality [43,66–69]. This seems particularly relevant for carotenoid (orange) and melanic (black) colours, which are thought to be costly to maintain in nature [69,70]. It is also possible that female preference for certain environmental-influenced colours such as orange could evolve as a 'pleiotropic' interaction with foraging behaviour (i.e., hypothesis of sensory bias; [71]. In contrast, maintenance of high variability in the less abundant colours is consistent with the role of negative-frequency selection by females for rare colour patterns [14,63,72,73]. This remarkable congruence across sites indicates that female preference (for honest signals and/or rare colours) likely plays a major role in shaping contemporary colour patterns in introduced populations (e.g., [31,43,44,48]).

On the other hand, however, there was no clear or statistically significant divergence in colour patterns between native high and low predation populations (Fig 4). These findings are therefore inconsistent with the classical “story” about how colour diverges between predation regimes. This could partly reflect the low power of our analyses (using population-level means only, and few studies), but also suggests that in general this colour polymorphism may be much less clear-cut than traditionally thought [12,16,21,27–29].

Contrary to our expectations, introduced low predation populations in Panama did not diverge from native high predation populations. For most colours, introduced populations were either intermediate or completely overlapping with both native low and high populations (Fig 4), and there were no statistically significant differences between any of these comparisons. This contradicts the expectation that introduced populations in Panama–being released from predation–would evolve to be significantly more colourful than native high predation populations. This suggests that colour evolution in the introduced range may be constrained by additional factors known to influence colour patterns in *P*. *reticulata*. Some of these factors include local environmental variation [12,18,32,57,69], available resources [17], low genetic variation [74,75] and low levels of plasticity [76,77]. Although we did not measure all of these factors in our study, a recent study has revealed low genetic variation in introduced guppy populations in Australia [45]. Furthermore, it is important to highlight that we found considerable variation in habitat quality across our study sites, ranging from the relatively clean Santa Monica Lake to the highly sewage-polluted Cristo Sacramado stream (Table 1).

The role of local environmental factors is interesting because colour variation in native populations of *P*. *reticulata* is traditionally thought to be determined by a dynamic interplay between female preference and male survival in low and high predation environments [12,16,78]. In the absence of predators, it is therefore reasonable to expect an amplification of colour polymorphism in males [30]; however, it is possible that additional environmental factors (e.g. multifarious selection; [47,79,80]) play a role in maintaining/constraining adaptive colour polymorphism when populations are removed from their native environments. Overall, the low consistency observed here suggests that additional studies of introduced populations are necessary to better understand the evolution of colour polymorphism in P. retic*ulata*, *partic*ularly in the face of novel environments.

### Caveats

One major potential limitation of our study is the uncertainty that surrounds the origin of our study populations. We suspect that most contemporary *P*. *reticulata* populations in Panama were founded by individuals released from pet stores or by pet owners, owing to the proximity of the sampling sites to human settlements, and the absence of any documented government stocking efforts in the past century. If this assumption is correct, the source populations are likely to have been artificially selected for highly variable colour patterns; a trait preferred by pet owners. Moreover, the source populations may conceivably have had very different initial levels of standing phenotypic and genetic variation, and different amounts of time to adapt to their novel habitats.

However, it is possible that female preference (in the absence of predators) in combination with local environmental factors (see above) could have easily overridden this ‘rearing effect’ in the novel environments–a reasonable assumption given the time elapsed since the first report of *P*. *reticulata* in Western Panama (~10 years), and the tendency for rapid evolution in the species [12,81–83]. In addition, is generally assumed that colonizing individuals represent a small subsample of the original population, which could reduce genetic and phenotypic variation and limit the establishment success of populations in introduced environments (i.e., founder effect [84–86]). Contrary to this expectation, our results suggest that founder effect has had little effect on male colour patterns in Panama, given the high level of variation found across sites and between introduced and native populations. However, we cannot rule out the possibility that these populations are being constantly replenished by the introduction of new individuals from pet stores or pet owners.

Our comparisons with published values from the native range also suffer from several limitations. First, the quantification of colour patterns by different authors has the potential to bias, or at the very least, add noise to our estimates. Second, our reliance on published, population-level data greatly reduced our statistical power and our ability to make inferences about how colour varies across the introduced vs. native range. Although some trends emerged (for instance that orange and black are consistently dominant), the high variability across populations suggest that additional studies with larger sample sizes are necessary to draw definitive conclusions. Future analyses will therefore benefit from the inclusion of individual-level colour data–instead of only population means–from studies of native populations as well as other introduced populations of *P*. *reticulata* around the world.

### Divergent or convergent evolution during biological invasions?

There is increasing interest in understanding the extent to which evolutionary changes may occur during the invasion process, and the role that adaptation may play in facilitating the establishment of invaders in novel environments (see reviews by [87,88]). In some cases, invaders rapidly diverge from the ancestral (native) condition, often in ways that enhance their dispersal capability and exacerbate their ecological impact. For example, invasive cane toads (*Bufo marinus*) appear to have evolved longer legs, which allow them to travel faster and has increased the rate of their spread five-fold since they were first introduced to Australia [89]. Nematode lungworm parasites that infect the cane toad also underwent rapid adaptive evolution–in life history traits–at the expanding range-edge [90]. In other cases, rapid evolution may also occur, but in a direction that parallels that observed in the native range, and does not necessarily increase invasibility. For example, Huey *et al*. [91] found that fruit flies, *Drosophila subobscura*, introduced to North America evolved an adaptive cline in wing size that closely paralleled that observed in their ancestral range in Europe. Further examples are needed to elucidate the contexts under which divergent (e.g. [89,90]) or convergent (e.g. [91]) evolution is more likely during biological invasions, and the ultimate consequences for ecological interactions with native species. We propose that, as a widely-introduced species with a demonstrated potential for rapid evolution [12, 81–83], guppies are an ideal model system to explore these questions further.

### Conclusion

Overall we found colour patterns that were both consistent and inconsistent with the classical paradigm of colour evolution in guppies. The same colours (orange, iridescent and black) that dominate in native populations were also the most dominant in our introduced populations in Panama. However, there were no clear differences between either introduced-low and native low- and high predation populations. These results are therefore only partially consistent with the traditional role of female preference in the absence of predators and suggest that additional factors might be influencing colour patterns in the introduced range of the species.

Understanding the role of these factors will be important for understanding the success of introduced species in novel environments, and the contexts under which variation in adaptive traits parallels (or not) variation in the native range. Future studies are necessary to determine the universality of the mechanisms that promote/maintain adaptive colour polymorphisms in nature, particularly in the face of anthropogenic disturbances.

## Supporting Information

S1 Fig Neighbour-Joining tree representing *P*. *reticulata* from different geographic locations. The tree is based on Kimura-2-Parameter distance for the COI barcode gene from specimens from Panama, and sequences obtained from GenBank. We used *Cnesterodon decemmaculatus* as an outgroup.
(EPS)

S1 Table Color variation across sites in introduced population of *P*. *reticulata* in Panama. Results from non-parametric Kruskal-Wallis rank tests examining variation in the number and relative area of colour spots across low-predation populations. K-W refers to the Kruskal-Wallis test statistic. Values in bold represent statistically significant differences.
(PDF)

S2 Table Population level estimates of colour patterns in *Poecilia reticulata*. The data represent population-level mean values for different colour patterns from native (Trinidad) and Introduced (Panama) populations. Data form native populations were obtained from the published literature.
(CSV)
