# Supplementary material for: Low Predictability of Colour Polymorphism in Introduced Guppy (Poecilia reticulata) Populations in Panama
Source: PLoS One. 2016 Feb 10;11(2):e0148040. doi: 10.1371/journal.pone.0148040 (PMC4749237; doi:10.1371/journal.pone.0148040)
Supplement: S1 Table — Results from non-parametric Kruskal-Wallis rank tests examining variation in the number and relative area of colour spots across low-predation populations. K-W refers to the Kruskal-Wallis test statistic. Values in bold represent statistically significant differences. (PDF) [file pone.0148040.s002.pdf]

S1 Table. Color variation across sites in introduced population of *P. reticulata* in Panama. Results from non-parametric Kruskal-Wallis rank tests examining variation in the number and relative area of colour spots across low-predation populations. K-W refers to the Kruskal-Wallis test statistic. Values in bold represent statistically significant differences.

	Number of spots			Relative area		
**Colour**	**Df**	**K-W**	***P***	**Df**	**K-W**	***P***
Black	3	3.89	0.273	3	24.34	**<0.001**
Iridescent	3	31.82	**<0.001**	3	20.75	**<0.001**
Orange	3	1.68	0.639	3	14.68	**0.001**
Yellow	3	16.93	**0.001**	3	15.39	**0.001**
Blue	3	20.18	**0.001**	3	31.36	**0.001**
Green	3	33.16	**<0.001**	3	28.79	**<0.001**
